# Work productivity loss and indirect costs associated with new cardiovascular events in high-risk patients with hyperlipidemia: estimates from population-based register data in Sweden

**DOI:** 10.1007/s10198-015-0749-y

**Published:** 2015-11-25

**Authors:** J. Banefelt, S. Hallberg, K. M. Fox, J. Mesterton, C. J. Paoli, G. Johansson, L.-Å. Levin, P. Sobocki, S. R. Gandra

**Affiliations:** 1 Quantify Research, Hantverkargatan 8, 112 21 Stockholm, Sweden; 2 Strategic Healthcare Solutions, LLC, Baltimore, MD USA; 3 LIME/Medical Management Centre, Karolinska Institute, Stockholm, Sweden; 4 Amgen Inc., Thousand Oaks, CA USA; 5 Department of Public Health and Caring Sciences, Uppsala University, Uppsala, Sweden; 6 Department of Medical and Health Sciences, Linköping University, Linköping, Sweden; 7 IMS Health, Stockholm, Sweden

**Keywords:** Cardiovascular disease, Hyperlipidemia, Indirect costs, Productivity, I12

## Abstract

**Objectives:**

To estimate productivity loss and associated indirect costs in high-risk patients treated for hyperlipidemia who experience cardiovascular (CV) events.

**Methods:**

Retrospective population-based cohort study conducted using Swedish medical records linked to national registers. Patients were included based on prescriptions of lipid-lowering therapy between 1 January 2006 and 31 December 2011 and followed until 31 December 2012 for identification of CV events and estimation of work productivity loss (sick leave and disability pension) and indirect costs. Patients were stratified into two cohorts based on CV risk level: history of major cardiovascular disease (CVD) and coronary heart disease (CHD) risk equivalent. Propensity score matching was applied to compare patients with new events (cases) to patients without new events (controls). The incremental effect of CV events was estimated using a difference-in-differences design, comparing productivity loss among cases and controls during the year before and the year after the cases’ event.

**Results:**

The incremental effect on indirect costs was largest in the CHD risk equivalent cohort (*n* = 2946) at €3119 (*P* value <0.01). The corresponding figure in the major CVD history cohort (*n* = 4508) was €2210 (*P* value <0.01). There was substantial variation in productivity loss depending on the type of event. Transient ischemic attack and revascularization had no significant effect on indirect costs. Myocardial infarction (€3465), unstable angina (€2733) and, most notably, ischemic stroke (€6784) yielded substantial incremental cost estimates (*P* values <0.01).

**Conclusions:**

Indirect costs related to work productivity losses of CV events are substantial in Swedish high-risk patients treated for hyperlipidemia and vary considerably by type of event.

## Introduction

Cardiovascular disease (CVD), with the usual underlying pathology of atherosclerosis, is a major cause of premature death worldwide, and a substantial source of disability [1]. The most common manifestation of CVD is coronary heart disease (CHD). CHD has been estimated to be the leading cause of disability in Europe, accounting for approximately 10 % of total disability-adjusted life years [2]. CHD has also been estimated to be the leading cause of premature permanent disability in the US labor force [1]. Consequently, CHD and other forms of CVD contribute extensively to the total cost of healthcare [3–6]. Although CVD is most prevalent among the older population, a substantial number of working-age patients are diagnosed [7]. Indirect costs due to lost productivity therefore account for an important part of the total costs of CVD [1]. In Europe, indirect costs associated with productivity losses have been found to correspond to 21 % of the total costs of CVD [8]. The American Heart Association have estimated that indirect costs accounted for 36 % of the total 1-year costs of CVD in the US in 2006 [9], and that CVD-related indirect costs were US $172 billion in 2010 [10].

While several studies have reported the large societal costs associated with CVD, there is a scarcity of studies that have focused specifically on costs related to new cardiovascular (CV) events. This is especially the case for indirect costs. The few available studies that have estimated indirect costs related to productivity losses due to CV events are either relatively old [11], have been limited by sample size [12], or have focused on the effect of specific medical interventions on productivity losses [13, 14]. In addition, many estimates have been based on questionnaires that are vulnerable to recall bias and often yield varied estimates of productivity losses even when administered in the same setting [6]. Furthermore, comprehensive estimates of indirect costs of CV events across the CV risk continuum of hyperlipidemia patients are lacking from the literature. Recent studies have started to address these issues, however [15].

The objective of this study was to estimate productivity loss and indirect costs associated with CV events in high-risk hyperlipidemia patients in Sweden. Productivity losses were estimated by CV risk level and by the following event types: myocardial infarction (MI), ischemic stroke (IS), revascularization, unstable angina pectoris (UA), heart failure (HF), and transient ischemic attack (TIA). By providing estimates of indirect costs associated with lost productivity due to CV events, stratified by risk level and by CV event type, this study fills important gaps in available evidence. Reliable and up-to-date estimates of costs of clinical events are essential in economic evaluation studies comparing standard care to novel health technologies and interventions aimed at lowering the incidence of these events.

## Methods

### Study design and population

This was a retrospective population-based cohort register study based on a matched control design, conducted using patient-level data. The primary data sources for the study were electronic medical records in Swedish primary care, three national compulsory health registers governed by the National Board of Health and Welfare, and the Swedish Social Insurance Register. Unique individual patient ID numbers were available for all data sources, allowing for linkage of individual patients between data sets. The linkage of de-identified individual patients was performed by the National Board of Health and Welfare. The study was approved by the Swedish Ethics Review Board.

The study population was defined as high-risk patients with hyperlipidemia, as elevation of low-density lipoprotein cholesterol levels is closely linked to the development of CHD [1]. Patients were identified for inclusion in the study population based on treatment with lipid-lowering therapy (LLT), as registered in the Prescribed Drug Register. This was deemed the most accurate way of identifying hyperlipidemia patients in Sweden as the diagnosis of hyperlipidemia is often made in primary care, where reporting and coding of diagnoses is not mandatory and therefore relatively poor. High-risk patients with a filled prescription of LLT during the period between 1 January 2006 and 31 December 2011 were included, if a second filled prescription followed within 6 months. The study inclusion date was defined as the date of the first of the filled prescriptions for LLT. High risk was defined as a history of major CVD or diagnosis of a CHD risk equivalent condition, based on patient diagnosis history from 10 years prior to and up until study inclusion. Patients who were not defined as high risk were excluded from the study population.

Patients with a history of major CVD and patients with a CHD risk equivalent condition were considered as separate cohorts, in order to quantify productivity loss across the continuum of high-risk hyperlipidemia patients. The two cohorts were considered together in the analysis of specific event types, however, due to sample size considerations. The cohorts were defined based on the National Cholesterol Education Program Adult Treatment Panel III guidelines [16] and the European Society of Cardiology guidelines [17]. The two cohorts were defined as follows:

1. History of major CVD cohort: prior revascularization or diagnosis of MI, UA, or ischemic stroke.
2. CHD risk equivalent cohort: patients not included in the history of major CVD cohort and with prior diagnosis of diabetes, peripheral artery disease, abdominal aortic aneurysm, TIA, or stable angina pectoris.

Patients of working age at time of study inclusion were observed from study inclusion until 31 December 2011 (exposure time) for identification of new CV events using the National Patient Register. Working age was defined as between 20 and 64 years of age. The date of the first new CV event following study inclusion was defined as the index date. A new event was defined as an inpatient primary diagnosis of acute MI, UA, ischemic stroke, HF, TIA, or any inpatient or outpatient procedure code indicating revascularization. Revascularization was defined as percutaneous transluminal coronary angioplasty (PTCA). A minimum of 60 days was required between events of the same type in order for them to be considered separate events. In addition, a minimum of 60 days was required between any event type and a revascularization in order for the revascularization to count as a separate event. Patients were followed for 1 year after the index date for estimation of productivity loss and associated costs.

Productivity loss was defined as the sum of net sick leave and net disability pension days, both of which are part of the Swedish social insurance system. All people who live or work in Sweden are automatically covered by the Swedish social insurance, which provides financial protection for families and children. Individual-level statistics are available in the Social Insurance Register on all sick leave episodes lasting longer than 14 days (start and end dates of all episodes and cause of sick leave) that are covered by the social insurance at an individual level. Short-term sick leave episodes (≤14 days) are the responsibility of the employer and are thus not captured in the register or in this study. Moreover, individual data are available on episodes of disability pension (“sickness compensation” for 30- to 64-year-olds and “activity compensation” for 19- to 29-year-olds). Disability pension may be either time-limited or permanent, and requires at least an estimated 25 % reduction in work ability that is expected to remain for at least 1 year. The National Social Insurance Agency is the sole administrator of sick leave and long-term disability in Sweden. The study design thus allowed for complete coverage of productivity loss in the selected sample population of the study (with the exception of sick leave episodes ≤14 days). Net days were used as sick leave and disability pension may be either complete or partial in Sweden. Net days for each sick leave and disability pension episode were calculated by multiplying the number of days by the level of compensation of the respective episode. Thus, 30 days of 50 % sick leave would thus result in a net 15 days of sick leave, for example. Weekends and holidays were included in calculations of sick leave and disability pension days, meaning that the maximum number of days lost was 365 per year.

The value of lost productivity was estimated using the human capital approach [18]. The human capital approach assumes that the cost of a person’s reduced productivity is equal to the amount an employer would be willing to pay for that production [18]. This was assumed to be the average gross salary plus employer contributions in Sweden. The average monthly pay was taken from the database of Statistics Sweden and was 27,600 SEK for women and 32,100 SEK for men in 2012 [19]. Employer contributions in Sweden are 31.42 % [20]. The average daily salary plus employer contributions was thus calculated to be 1192 SEK for women and 1386 SEK for men. Note that these figures include weekends and holidays, ensuring that indirect costs were not overestimated by including weekends and holidays in the calculations of sick leave and disability pension days. The difference in wages of men and women was taken into account when calculating indirect costs by adjusting for the gender distribution of patients. All costs were inflated to 2014 SEK using publicly available inflation data and converted to 2014 EUR (conversion rate: 1 EUR = 9.27 SEK).

### Statistical analysis

Patients without a new CV event between study inclusion and 31 December 2011 (controls) were matched to patients with a new CV event (cases) within the same CVD risk cohort using propensity score matching based on a 1:1 match without replacement. The covariates used in the matching were measured at time of study inclusion. Controls were then assigned the same index date as that of their matched cases. The matching covariates were: age; gender; Charlson comorbidity index based on diagnoses during the 5-year period prior to study inclusion; number of hospital visits related to CV events during the 1-year period prior to study inclusion; and days of hospitalization related to CV events during the 1-year period prior to study inclusion. In addition, a case was matched to a control only if their study inclusion date occurred during the same calendar year. The propensity score was estimated using logistic regression and the caliper approach was used for matching. The caliper method uses a tolerance level of the maximum propensity score distance to avoid the risk of bad matches. The maximum propensity score distance was set to one-fourth of the standard deviation of the propensity score. The quality of the matching was evaluated by investigating the standardized differences between cases and matched controls in the variables used in the matching. An ex-ante limit of 10 % in standardized differences was set for all variables used in the matching.

A difference-in-differences (DiD) approach was employed to obtain estimates of productivity loss and indirect costs associated with new CV events. Productivity loss was calculated for each case and each corresponding matched control during the 1-year period prior to index event (pre period) and during the 1-year period following index event (post period). The primary outcome was defined as the difference in the post versus pre difference in productivity loss between cases and propensity score matched controls.

$${\text{DiD}}\,{\text{estimate}} = ([{\text{Post}}\,{\text{period}}_{\text{Case}} {-} {\text{Pre}}\,{\text{period}}_{\text{Case}} ] - [{\text{Post}}\,{\text{period}}_{\text{Control}} {-}{\text{Pre}}\,{\text{period}}_{\text{Control}} ])$$

The DiD approach was used in order to control for potential confounders and limit bias in the obtained estimates. The within-subject comparison of cases before and after the event controlled for all time-invariant unobservable and observable characteristics by using each case as its own control. Further, the within-subject comparison of controls before and after index was used for correcting for secular trends and shocks to the outcome variable that would otherwise bias the pre versus post event comparison of cases. Controlling for such temporal effects is crucial when studying productivity losses in Sweden as changes have been made to the social insurance system in recent years [21], and sick leave and disability pension are likely correlated with the business cycle. In addition, the matching of cases and controls based on observable characteristics was performed to ensure that secular trends and shocks that affect productivity losses impact on cases and controls equally, by constructing the control cohort to be similar to cases in terms of observable characteristics.

Case patients were followed for 1 year following the index event in parallel to their matched controls, and were censored when either they or their matched control died, whichever came first. In addition, matched patient pairs were censored at time of a second event in cases following index in order to ensure that the estimates of productivity loss and costs of the index event were not influenced by productivity losses and costs related to subsequent events. Furthermore, patient pairs were removed from the analysis if the control was no longer alive at index date.

The non-parametric Wilcoxon signed-ranked test was used to test for statistical significance in differences in costs. Differences were considered statistically significant if *P* values were less than 0.05.

All data management and statistical analysis was performed using MySQL and Stata 12.

## Results

### Patient attrition and characteristics

**Fig. 1:**
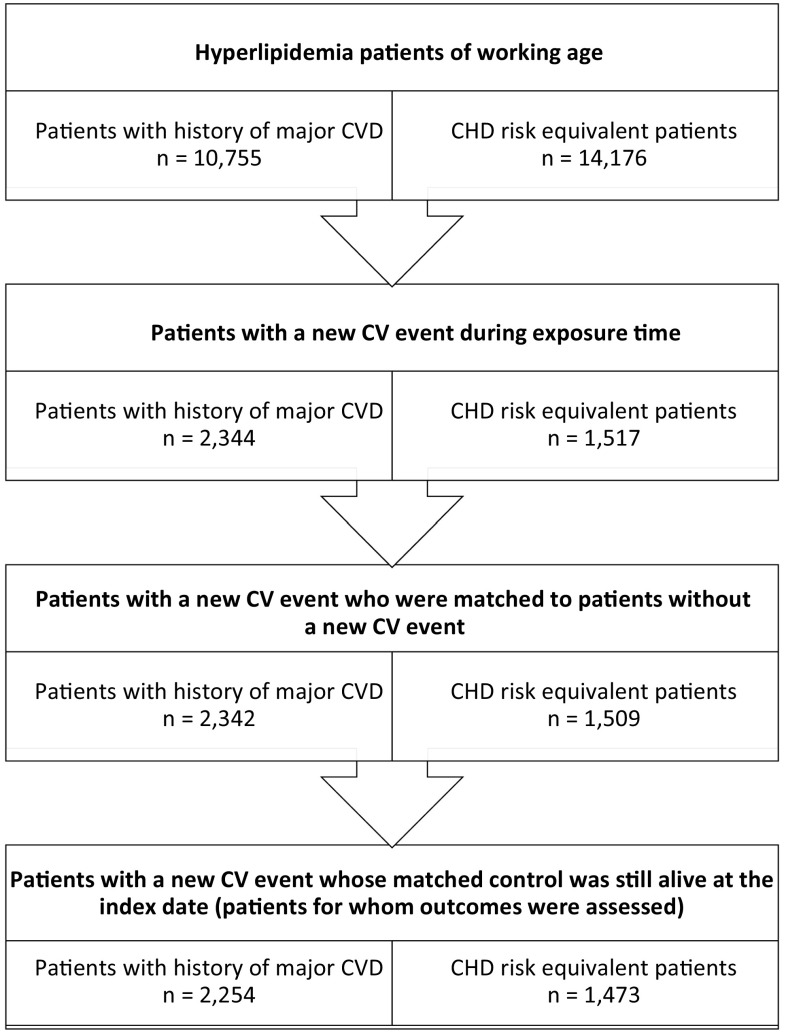
Patient attrition

A total of 24,931 working age patients treated for hyperlipidemia were identified for inclusion in the study and stratified to one of the two cohorts. Of the included patients, 10,755 (43 %) were stratified to the history of major CVD cohort and the remaining 14,176 (57 %) patients were stratified to the CHD risk equivalent cohort. Of these, 2344 (22 %) and 1517 (11 %) patients, respectively, experienced a new CV event following study inclusion. Outcomes were assessed only in cases matched to a control who was still alive at the index date. Outcomes were thus assessed in 2254 cases and equally many controls with a history of major CVD, and in 1473 CHD risk equivalent cases and equally many CHD risk equivalent controls. Figure 1 illustrates the stepwise inclusion and exclusion of patients, by cohort, following this procedure.

Standardized differences were lower than the ex-ante limit of 10 % for all of the variables included in the matching at study inclusion. The characteristics of the two cohorts at index date are presented in Table 1. In both cohorts, cases and controls were balanced at index date in terms of age and gender. Cases were noticeably more comorbid than controls in both cohorts, however, despite the fact that the Charlson index was used in the matching of patients at time of study inclusion. This indicates that the prevalence of comorbid conditions increased more in cases between time of study inclusion and index date than in controls.

**Table 1:** Patient baseline characteristics at index date for cases (matched patients with new cardiovascular events) and controls (matched patients without new cardiovascular events), for both cohorts.* CVD* Cardiovacular disease,* MI* myocardial infarction

Patient baseline characteristics	History of major CVD cohort (*n* = 4508)		CHD risk equivalent cohort (*n* = 2946)	
	Cases (*n* = 2254)	Controls (*n* = 2254)	Cases (*n* = 1473)	Controls (*n* = 1473)
Age [mean (SD)]	56.49 (5.95)	56.40 (5.86)	56.88 (5.89)	56.88 (5.93)
Female (%)	24	22	32	31
Charlson comorbidity index [mean (SD)]	2.00 (1.95)	1.46 (1.74)	2.27 (2.15)	1.77 (1.88)
Myocardial infarction (%)	36	24	2^a^	0
Diabetes mellitus (%)	24	18	49	45
Cerebrovascular disease (%)	22	20	11	6
Congestive heart failure (%)	18	7	14	5
Chronic pulmonary disease (%)	10	7	13	8
Peripheral vascular disease (%)	9	7	12	7
Moderate severe renal disease (%)	7	4	10	6
Malignancy (%)	6	6	6	7
Connective tissue disease (%)	4	3	4	3
Ulcer disease (%)	3	2	3	2

^a^Patients with no primary diagnosis of MI prior to study inclusion but who had an MI listed as a secondary diagnosis between study inclusion and index event

The two cohorts were similar in terms of age at index, while a larger percentage of CHD risk equivalent patients were female compared to patients with a history of major CVD. The CHD risk equivalent patients were also more comorbid compared to their history of major CVD counterparts.

### Productivity loss by risk level

Table 2 presents productivity losses in both cohorts. The corresponding indirect costs are presented in Table 3. The sum of net sick leave and net disability pension days in the history of major CVD cohort increased by 4.35 in cases and decreased by 10.70 in controls between the pre and post periods. The DiD estimate of total days lost due to the event during the 1st year after the event was thus 15.05, corresponding to indirect costs of 2210 EUR. In the CHD risk equivalent cohort, cases experienced an increase of 9.67 days lost between the two periods. Controls experienced a decrease of 12.11 days during the same period, yielding a DiD estimate of days lost due to the event during the year after the event of 21.78. This corresponded to indirect costs of 3119 EUR.

**Table 2:** Productivity losses in cases and controls, both cohorts

	*n*	Mean days of sick leave + disability pension (SE)		
		Pre-period	Post-period	Difference
History of major CVD cohort				
Cases	2254	154.35 (3.38)	158.70 (3.46)	4.35
Controls	2254	119.94 (3.28)	109.24 (3.64)	−10.70
Difference				15.05*
CHD risk equivalent cohort				
Cases	1473	149.81 (4.25)	159.48 (4.40)	9.67
Controls	1473	107.10 (3.93)	94.99 (4.25)	−12.11
Difference				21.78*

* *P* < 0.01

**Table 3:** Indirect costs of cases and controls, both cohorts

	*n*	Mean cost of sick leave + disability pension in EUR (SE)		
		Pre-period	Post-period	Difference
History of major CVD cohort				
Cases	2254	21,994 (483.29)	22,667 (496.70)	673
Controls	2254	17,135 (470.10)	15,598 (521.40)	−1537
Difference				2210*
CHD risk equivalent cohort				
Cases	1473	21,023 (598.16)	22,460 (617.51)	1437
Controls	1473	14,993 (551.87)	13,311 (600.38)	−1682
Difference				3119*

* *P* value <0.01

### Productivity loss by type of event

**Fig. 2:**
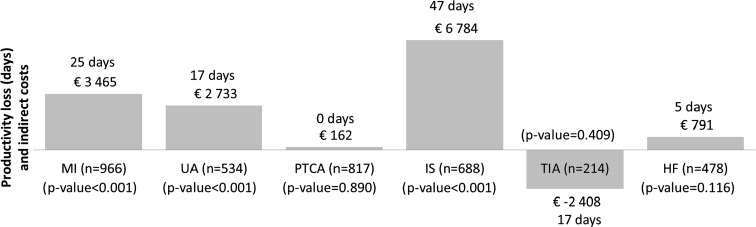
Difference-in-differences (DiD) estimate of productivity loss and indirect costs by index cardiovascular (CV) event type (both cohorts combined). *MI* Myocardial infarction, *UA* unstable angina, *PTCA* percutaneous transluminal coronary angioplasty (revascularization), *IS* ischemic stroke, *TIA* transient ischemic attack, *HF* heart failure

Productivity loss and associated indirect costs were also estimated by CV event type. These analyses were performed for both strata combined. Figure 2 presents DiD estimates of productivity loss and associated indirect costs by index CV event type. MI was the most common event (*n* = 966), followed by revascularization (*n* = 817), ischemic stroke (*n* = 688), UA (*n* = 534), HF (*n* = 478), and TIA (*n* = 214). The effect on productivity losses varied substantially among these event types. The largest impact was observed in patients experiencing ischemic stroke (47 days; 6784 EUR). MI and UA also lead to significant increases in productivity loss during the year following the event (25 and 17 days, corresponding to indirect costs of 3465 EUR and 2733 EUR, respectively). The effects of revascularization, TIA, and HF were statistically insignificant.

## Discussion

The objective of this study was to estimate productivity loss and indirect costs associated with CV events in high-risk hyperlipidemia patients in Sweden. Estimates were generated for two distinct CV risk levels, as well as by type of event. There is a scarcity of available data on productivity losses and related indirect costs specifically due to CV events. Further, there is a lack of understanding of the effects of CV events across the continuum of patients at elevated risk of experiencing such events and of how these effects may vary depending on the type of event. Zethraeus et al. [11] estimated the direct and indirect costs of CHD and stroke in Sweden and presented estimates by type of clinical event. However, the study is relatively old and is limited by its sample size (*n* = 12 for each event type in the calculations of indirect costs). In addition, indirect costs were calculated by multiplying the number of weeks that the individual worked during a year by the mean number of hours of work per week and the labor costs for wage earners in mining and manufacturing. This differs from the human capital approach used to calculate indirect costs in this study. Lindgren et al. [12] similarly estimated indirect costs associated with cardiovascular events. However, the Lindgren study was also limited by sample size (*n* = 60) and estimated productivity losses and resulting indirect costs through the use of a questionnaire. The estimates are thus vulnerable to recall bias. Further, estimates of indirect costs were not presented by type of event. A recent study by Song et al. [15] addresses many of these issues by providing up-to-date estimates of indirect costs by event type using a large sample of patients with hyperlipidemia. The study controlled for confounders by matching patients with and without events using propensity score matching. However, differences in unobservable characteristics between cases and controls were not controlled for. In contrast, the DiD approach used in this study controls for all time-invariant observable and unobservable characteristics.

The stratification of patients by risk level and by type of event contributes to filling important evidence gaps in the literature and is an important strength of this study. The high representativeness and the quality of the data used in the analyses are also strengths. Sweden is well-known for having excellent registers with a high degree of validity and completeness that allow for generating real-world evidence of high quality. Furthermore, a unique feature of this study is that estimates of the impact of CV events on productivity loss were generated by a DiD approach. The DiD approach controls for time-invariant observable and unobservable factors, as well as secular trends and shocks in the outcome variables over time that are common to cases and controls. Matching on observable characteristics was performed in order to ensure that trends and shocks in the outcome variables were common to cases and controls. The estimates should be viewed as conservative, as the analysis does not include short-term sick leave episodes and patients were censored at time of a second event following index event.

A limitation of the study is the lack of a natural index date for controls, as controls by design did not experience any events during exposure time. Cases and controls were therefore matched based on their characteristics at study inclusion rather than at start of follow-up (i.e. index date), when the comparison of cases and controls starts. Controls were then assigned the same index date as that of their matched cases. This discrepancy may have led to matched pairs being less comparable at start of follow-up than they were at the time of matching. There was some evidence of this, as cases had a higher Charlson comorbidity index at index date compared to their matched controls and baseline productivity losses of cases were generally higher than in controls.

It should also be noted that not all data on productivity loss were available for analysis. Short-term sick leave episodes (≤14 days) are the responsibility of the employer in Sweden and are therefore not recorded in the Social Insurance Register. Thus, such episodes could not be included in the analysis. The first 14 days of all recorded sick leave episodes (i.e., those longer than 14 days) are recorded in the register, however. Even so, the study underestimates total productivity losses and associated costs of CV events due to this limitation. The finding that revascularization did not lead to an increase in productivity loss is likely due to the fact that short-term sick leave episodes are not captured in the study. Revascularization differs from the other events in that it is a procedure rather than a medical condition. Revascularizations can therefore be planned, meaning that the sick leave following the procedure can also be planned. Sick leave episodes following planned revascularizations are therefore often shorter than the 15 days required for the episode to be recorded in the Social Insurance Register. Caution is thus warranted when interpreting the estimates of the effect of revascularization.

Further, the observation time of patients was shorter during the period following index event compared to the pre-period used as a baseline, due to patients dying during the period following index event as well as being censored at time of a second event. This in turn results in productivity losses being lower during the post-index periods than they otherwise would have been, as patients will by definition experience no productivity losses once they are deceased or censored. In addition, since pre-index levels of productivity losses were higher in cases than in controls, this decrease was greater in cases than in controls in absolute terms. This further impacts the DiD estimates obtained, in the case of TIA even leading to a negative estimate (i.e. indicating that a TIA is associated with cost savings). At the same time, cross-sectional comparisons of cases and controls would be insufficient, due to the fact that pre-index levels of productivity losses were higher in cases than in controls. Similarly, within-subject comparisons do not control for changes in the outcome variable that would have occurred even in the absence of an event.

In conclusion, this study demonstrates that CV events in Sweden are associated with substantial short-term productivity losses and consequent indirect costs in high-risk working-age patients treated for hyperlipidemia, highlighting the unmet needs of these patients. Large productivity losses prior to index event were also demonstrated, as patients had between 107 and 154 mean days lost during the year prior to index event. This burden significantly impacts employers and society as a whole. There is thus large potential for reducing productivity loss and associated costs from new interventions that would help in primary and secondary prevention of CV events. However, the effect on productivity losses varies considerably depending on the type of event, with ischemic stroke associated with by far the largest indirect costs of the investigated event types.
